# Supplementary material for: Maternal depression in Latinas and child socioemotional development: A systematic review
Source: PLoS One. 2020 Mar 12;15(3):e0230256. doi: 10.1371/journal.pone.0230256 (PMC7067456; doi:10.1371/journal.pone.0230256)
Supplement: S2 Table — (DOCX) [file pone.0230256.s002.docx]

**S2 Table. Cognitive Outcomes (n = 4)**

| Study | Purpose | Study Design | Sample/Setting | Measure of Maternal Depression and data collection points | Measure of Cognitive outcome and data collection points | Country of origin and any acculturation data | Analysis and covariates | Results |
| --- | --- | --- | --- | --- | --- | --- | --- | --- |
| **Prenatal and Infancy** | | | | | | | | |
| Aguilar Schall,  (2011) | To examine the impact of postpartum depression on 12-month old infant neurodevelopment | Longitudinal:  13 and 26-weeks gestation, at delivery, six and 12 months postpartum | n=350  The Center for the Health Assessment of Mothers and Children of Salinas birth cohort (CHAMACOS)  Mexican-American birth cohort studying effects of pesticides  Low-income mother-infant dyads  Maternal age:  >18  Mean (M)=26.5 Standard Deviation (SD)=5.3 | CES-D >16 at 12 months postpartum | Infant neurodevelopment measured at 12 months:  Expressive communication: PLS-EC-III  Auditory communication: PLS-AC-III  Memory, problem-solving, language, psychomotor, and personal-social abilities measured by BSID-II, comprised of three scales:  -MDI (mental)  -PDI (psychomotor) -Behavior Rating Scale | Mexican-origin  11% born in U.S.  89% born in Mexico | Analysis: standard parametric regression, various “machine learning tools” and data adaptive techniques to determine optimal models    Intermediate variables: HOME, breastfeeding duration  Covariates: maternal age, maternal education, years in the U.S., language spoken at home, marital status, presence of father at home, child's gender, number of other children in home, PPVT-III score, poverty level, infant cared for outside of home, social support score, child’s age, and psychometrician performing evaluations | Maternal depression was significantly associated with lower PLS-EC (4.7 [95% CI: 1.8, 7.5], p=.001) and PLS-AC (2.9 [95%CI: 0.2, 5.5], p=.04) scores when using simple association analysis  Association between lower PLS-EC scores and maternal depression retained significance when incorporating intermediate variables and covariates into models: -2.82 (95%CI: (-5.61, -0.04), p-=0.05). Results remained significant regardless of the tools used to calculate models.  No significant association between maternal depression and other child outcomes |
| Cabrera  et al.,  (2006) | To examine how mother – infant interactions, father engagement, and infant cognition vary based on country of origin, socioeconomic status, and English language proficiency | Cross-sectional:  Data collected when infant was 9 months old | n=1,099  Latino children living with both biological parents at 9 months of age  from the Early Childhood Longitudinal Study – Birth Cohort  Maternal age:  M=27.54  SD=5.70  15-40+  Child age:  M=10.21 months  SD=1.41 | CES–D Short Form>16  when infant was 9 months old  Maternal sensitivity at 9 months:  NCATS | Infants’ cognitive development at 9-months:  BSF–R  (mental subscale) | 67%: Mexican-American  33%: Puerto Rican, Central American, and Dominican  Acculturation measured by:  Language proficiency  Maternal English proficiency was significantly correlated w/ maternal interactions ß=.17, p <.001 but paternal English proficiency was not | Analysis: multiple linear regression  Covariates: SES, child age and gender | Depressive symptoms were not associated with mother–infant interaction.  Maternal depressive symptoms were negatively associated with higher cognitive test scores, ß= .07, p=.04, but paternal depression was not.  Higher maternal interaction scores were associated with higher cognitive test scores, ß=.09, p>.05, but paternal interaction was not |
| **Preschool** | | | | | | | | |
| Cycyk et al.,  (2015) | To examine the impact of maternal depressive symptoms and social support on bilingual children’s language development | Longitudinal:  Each Fall and Spring of children’s two years in Head Start before entering kindergarten | n=83  Low-income bilingual mother-child dyads enrolled in a larger longitudinal study examining bilingual language development  Maternal age: M=25.99 SD=5.16  19-48  Child age: M=43 months SD=4 | CES-D>16  at each Fall and Spring of the children’s two years in Head Start  Maternal social support: PSS-Fa (family) and PSS-Fr (friends) at the Fall of each year | Each Fall and Spring of children’s two years in Head Start (English Immersion) before entering kindergarten (approximately 6 months part)  Spanish Receptive vocabulary: TVIP  English receptive vocabulary: PPVT-III  Spanish expressive oral and auditory comprehension abilities: SPLS-3  English expressive oral and auditory comprehension abilities: TELD-3 | Puerto Rican descent:  1^st^ generation: 18.8%  2^nd^ generation: 55.3%  3^rd^ generation: 22.4%  4^th^ generation: 3.5%  All children exposed to Spanish from birth:  31% Spanish only  35% equal amounts of Spanish and English  44% more English than Spanish | Analysis: growth curve modeling  Covariates: child’s exposure to English and Spanish at home tested in models and deemed non-confounding | Maternal depression was significantly negatively associated with the growth of TVIP (Spanish receptive vocabulary) scores: *t* (235.64) =-1.99, p=.047)  No significant association between maternal depression and other language scores |
| Palermo et al., (2018) | To examine the relationship between economic hardship during infancy, “maternal mental health problems”, “maternal positive parenting behaviors”, and Latino children’s socio-behavioral difficulties and academic skills prior to kindergarten entry- using a culturally integrated Family Stress Model; do acculturation levels moderate the pattern of associations? | Longitudinal: children at 4, 14, 24 and 36 months, and half a year before kindergarten | n=714  Early Head Start Research and Evaluation Project (EHSREP) Low-income Latina mothers  Maternal age:  84.6%>18  M=24  SD=6  13-43  Child age at study entry: M=4 months | At 14 months,  “maternal mental health problems”  quantified by Depression: CES-D, Parenting Stress: PSI-SF, Perceived Control: Pearlin Mastery Scale  Maternal sensitivity at 14 months:  Three Bag Task | Half a year before kindergarten:  -Academic skills: Woodcock-Johnson Achievement III or Woodcock Muños-Revised  Subscales: Letter Word Identification and Applied Problems | 82% Mexican-American; 59% foreign-born; 9% Central American; 6% Puerto Rican  At 24 months old, mother’s acculturation levels measured by generational status, English use preference, and proficiency: Multi-cultural Acculturation Scale, Picture Vocabulary subscale of the Woodstock-Johnson Achievement III  Positive association between maternal acculturation and positive parenting behaviors (ß=.22, SE=.06, p<.001) | Analysis:  Structural equation modeling  Covariates:  - child’s gender, maternal education, family structure  -At 14 months: cognitive ability measured by MDI Bayley | Positive association between economic hardship and maternal mental health problems (ß=.50, SE=.08, p<.001)  Negative association between maternal mental health problems and maternal positive parenting behaviors (ß=-.16, SE=.06, p=.008)  Maternal mental health problems and maternal positive behaviors significantly mediated the association between economic hardship and children’s academic skills (ß=-.05, SE=.02, p=.019  Relationship patterns did not vary by acculturation levels |

*Note.* BSID-II: Bayley Scales of Infant Development, Second Edition; HOME: Home Observation for the Measurement of the Environment; NCATS: Nursing Child Assessment Teaching Scale; MDI Bayley: Bayley II Mental Developmental Index; PLS-AC: Pre-School Language Scale – Auditory Comprehension; PLS-EC: Pre-School Language Scale – Expressive Communication; PSI-SF: The Parenting Stress Index – Short Form; PPVT-III: Peabody Picture Vocabulary Test-III; SPLS-3: Spanish version of the Preschool Language Scale-Third Edition; TELD-3: Test of Early Language Development-Third Edition; TVIP: Test de Vocabulario en Imágenes Peabody
