# Supplementary material for: Maternal depression in Latinas and child socioemotional development: A systematic review
Source: PLoS One. 2020 Mar 12;15(3):e0230256. doi: 10.1371/journal.pone.0230256 (PMC7067456; doi:10.1371/journal.pone.0230256)
Supplement: S1 File — (PDF) [file pone.0230256.s003.pdf]

Maternal depression in Latinas and children socio-emotional and cognitive outcomes: a systematic review

Hudson Santos, Rebeca Harris

Citation

Hudson Santos, Rebeca Harris. Maternal depression in Latinas and children socio-emotional and cognitive outcomes: a systematic review. PROSPERO 2019 CRD42019128686 Available from: https://www.crd.york.ac.uk/prospero/display_record.php?ID=CRD42019128686

Review question

During the foundational years for neuroplasticity from the prenatal period to early childhood (0-5 years), how does maternal depression in Latina mothers relate to their children's socioemotional and cognitive outcomes?

i. P: Low-income children who have a Latina mother with depression (during any period from pregnancy to early childhood, 0-5 years old)
ii. I: N/A
iii. C: child's exposure to maternal depression versus no exposure
iv. O: child's socioemotional and cognitive development

Searches

Studies in this systematic review were identified through both manual searches and the following electronic databases: PubMed, CINAHL, and PsycINFO. A search string intended to comprehensively capture studies examining the impact of maternal depression on young children's socioemotional and cognitive outcomes within the Latino population was applied to all three databases: Postpartum OR Depression OR Depressive Disorder OR Depress* AND Postpartum Period OR Postnatal Care OR Prepartum OR pre-partum OR prenatal OR pre-natal OR antenatal OR perinatal OR peripartum OR postpartum OR post-partum OR postnatal OR post-natal OR puerperium OR parturition OR pregnanc* OR pregnant or mother* OR newborn* AND Hispanic Americans OR Latina* OR Latino* OR Hispanic* AND child* OR infant OR toddler OR preschooler OR pediatric AND develop* OR behavior* OR cognitive OR emotion* OR conduct OR language OR temperament OR socio* OR regulation OR internalizing OR externalizing OR psych*. Reference lists from relevant literature were manually searched for any additional eligible studies. The search was run through to October 19, 2018. Studies could be in English or Spanish.

Search strategy

Types of study to be included

There is no restriction of the type of study designs. Studies were selected using the four-phase process for systematic reviews recommended by The PRISMA Group

Condition or domain being studied

Studies examining the impact of maternal depression on socioemotional and cognitive child development within the Latino population will be systematically reviewed. Given the significant Hispanic population growth in the U.S. and the high prevalence of depression in Latina mothers, this review has important implications for policy and both maternal and pediatric mental health. In this review, we will look at the effects of maternal depression in Latina mothers on children socioemotional and cognitive outcomes in the first five years of life.

Participants/population

To be considered for inclusion, the study needed to assess the relationship between maternal depressive

symptoms and children's socioemotional or cognitive outcomes within Latina mother-child dyads living in the United States. For the current study, "Latina" refers to a female of Latin American origin or descent (e.g., Mexico, Cuba, Puerto Rico, South and Central America), regardless of race. Moreover, socioemotional development encompasses a child's emerging capacity for the emotional, behavioral, and social competencies underlying early childhood mental health. Further inclusion criteria included (i) young children exposed to maternal depression at any point prenatally until aged five years old, allowing us to focus on foundational sensitive early developmental windows; (ii) adult mothers aged at least 18 years old; and (iii) specified economic status. Studies examining subpopulations with confounding secondary diseases or conditions that would inherently alter maternal depressive symptoms or socioemotional and cognitive outcomes (e.g., HIV, natural disasters, or developmental delays) were excluded. There were no time restrictions applied to ensure comprehensive results.

Intervention(s), exposure(s)

Not applicable

Comparator(s)/control

Not applicable

Context

Main outcome(s)

Children socioemotional and cognitive outcomes measures by clinical assessment or maternal report. Socioemotional development encompasses a child's emerging capacity for the emotional, behavioral, and social competencies underlying early childhood mental health.

Timing and effect measures

First five years of life

Additional outcome(s)

None

Timing and effect measures

Not applicable

Data extraction (selection and coding)

Data from included literature will be extracted onto a template which included study purpose, first author, publication year, study design, sample characteristics, maternal depression measures and data collection points, socioemotional or cognitive outcome measures and data collection points, main analytical approach and covariates, and main results. When available, country of origin and any acculturation data was extracted. A second reviewer evaluated the extracted data for thoroughness.

Risk of bias (quality) assessment

Study quality will be assessed by two reviewers following the guidelines established in the National Heart, Lung, and Blood Institute (NHLBI) Quality Assessment Tool for Observational Cohort and Cross-Sectional Studies.

Strategy for data synthesis

Data from included literature is being extracted onto a template which included study purpose, authors, publication year, study design, sample characteristics, maternal depression measures and data collection points, socioemotional or cognitive outcome measures and data collection points, main analytical approach and covariates, and main results. When available, country of origin and any acculturation data will also be extracted. A second reviewer is evaluating the extracted data for thoroughness. This synthesis will be

presented in table format, and a narrative synthesis will be used to describe the results. In addition, we are performing a quality assessment of the studies included by following the guidelines established in the National Heart, Lung, and Blood Institute (NHLBI) Quality Assessment Tool for Observational Cohort and Cross-Sectional Studies (NHLBI, 2014). The quality assessment results will be presented in Table format, and a narrative description will be provided. This process will be performed by two members of our team, and discrepancies are resolved through discussion involved the entire team.

Analysis of subgroups or subsets
None planned

Contact details for further information
Hudson Santos
hsantos@unc.edu

Organisational affiliation of the review
University of North Carolina at Chapel Hill

Review team members and their organisational affiliations
Dr Hudson Santos. University of North Carolina at Chapel Hill
Rebeca Harris. University of North Carolina at Chapel Hill

Type and method of review
Narrative synthesis, Systematic review

Anticipated or actual start date
01 September 2018

Anticipated completion date
01 May 2019

Funding sources/sponsors
None

Conflicts of interest

Language
English, Spanish

Country
United States of America

Stage of review
Review Ongoing

Subject index terms status
Subject indexing assigned by CRD

Subject index terms
Child; Cognition; Depression; Depressive Disorder; Emotions; Hispanic Americans; Humans

Date of registration in PROSPERO
06 August 2019

Date of publication of this version
06 August 2019

Details of any existing review of the same topic by the same authors

Stage of review at time of this submission

Stage	Started	Completed
Preliminary searches	Yes	Yes
Piloting of the study selection process	Yes	Yes
Formal screening of search results against eligibility criteria	Yes	Yes
Data extraction	Yes	No
Risk of bias (quality) assessment	Yes	No
Data analysis	Yes	No

Versions

06 August 2019

PROSPERO
This information has been provided by the named contact for this review. CRD has accepted this information in good faith and registered the review in PROSPERO. The registrant confirms that the information supplied for this submission is accurate and complete. CRD bears no responsibility or liability for the content of this registration record, any associated files or external websites.
