# Supplementary material for: Maternal depression in Latinas and child socioemotional development: A systematic review
Source: PLoS One. 2020 Mar 12;15(3):e0230256. doi: 10.1371/journal.pone.0230256 (PMC7067456; doi:10.1371/journal.pone.0230256)
Supplement: Attachment — Submitted filename: Review PLOS.docx [file pone.0230256.s004.docx]

Thank you for opportunity to review the manuscript "Maternal Depression in Latinas and child socioemotional and cognitive development: A systematic review.” The authors are addressing a very important topic with implications for psychological care for Latino populations for whom psychological interventions have failed to serve well historically. This manuscript has a number of primarily methodological strengths such as pre-registering the study, using the PRISMA guidelines, and coding to data quality which are all very important. However there are several concerns primarily in the introduction in terms of setting up the aims and scope of the review that detract from the manuscripts potential.

General comments

1. Use consistent terms for your constructs (many different terms for the outcome throughout) and define your constructs upfront and all that they encompass for clarity. There are many examples of this but for instance you say you at just looking at 0-5 years of age but cite a handful with kids older than 5 and say you are only looking at maternal depression but cite a handful that combine stress and depression or depression and other psychopathology.
2. Mediation and moderation are distinct conceptually and have different implications so the many instances of grouping them together is misleading. Set them up separately and interpret them separately since they tell us different things.

Introduction

1. My main comment is that your outcome (essentially all biological social and psychological aspects of development) is far too broad to make a cohesive argument and defined differently in different places. You look at child neurodevelopment, language development, social development, emotion regulation and self regulation, psychophysiology (RSA, EEG, ANS reactivity), attachment and psychological problems (e.g. externalizing). It would be very hard to review the background literature on all of these things adequately in the introduction which makes for a premise that is not well set up. I would strongly recommend narrowing your focus and in particular getting rid of the one study here and there that looks at a different construct entirely than the others (e.g. attachment and psychophysiology). I would recommend focusing on psychological outcomes (social behavior and externalizing) and closely related constructs (e.g. emotion regulation) since the studies seem to overwhelmingly focus on this, its much easier to build a cohesive theory around this, and you can clearly define the literature and your construct for readers to begin with.
2. Related to defining your constructs clearly what is meant by economic hardship you go on to describe community violence, overcrowded housing, poverty (defined as below the poverty line I assume) as variables in the studies so make it clear these are all proxy for poverty that you will be considering under the umbrella of “economic hardship.”
3. Restructuring thoughts: Your paper would benefit from an integrated conceptual model presented early on (a figure would be helpful), then the evidence of the model in white families, then the evidence in LatinX families and the need for research in LatinX families. Instead you highlighted different pieces from different angles (eg. Maternal sensitivity paragraph, FSM paragraph, and acculturative stress paragraph). It seems that your full model is that acculturative stress and economic stress (poverty) leads to maternal depression which impacts poor child outcomes through compromised parenting. Then that we should focus on Latina moms because they disproportionately experience acculturative stress and poverty. You should also mention that not all of the studies cover all pieces of the model but they all have the maternal depression and child outcomes piece in Latina samples.
4. Make the argument or mothers vs. fathers. The argument is maternal depression has a robust impact on child outcomes prenatally an there are less studies on fathers I assume but this needs to be explicit.
5. You should comment on the immigrant paradox literature explicitly in the argument for Latinas specifically section.
6. Line 53: comment on depression rates in Latinx populations specifically
7. Developmental argument: I would recommend adding to your developmental windows section talking about why you chose 0-5 year olds specifically (e.g. include the age ranges in the studies you are citing) given that this is never said until the last sentence of the introduction. What is the evidence (not just the theory) that this range is integral? For example, you could cite here evidence that intervening on child outcomes for 0-5 year olds with depressed mothers improves child psychological outcomes long term. Also explain why you use 5 as a cutoff and not earlier? Attachment theory may also be helpful to augment this developmental argument. I love the developmental figure though, that was very helpful.
8. Relatedly don’t review literature on adolescent outcomes (line 112) if your focus is 0-5 years of age, this is inconsistent with your argument for focusing on 0-5.
9. Biology is not “permanent” as you say in line 70, there is overwhelming evidence of plasticity in biological responses which is hopeful for interventions.

Methods

1. Your methods were very strong on the whole and my main comment is taking out the less relevant articles as stated above since it detracts from being able to make tangible and focused conclusions but also have some minor comments below.
2. Line 157: did you exclude for psychological co-morbid diagnoses (e.g. anxiety etc)?
3. It may be helpful to describe the last date of searching databases so future researchers will know where to pick up for later reviews. For example “ we reviewed all studies before June of 2019.”
4. Line 233: brief description of the three bag and two bag paradigms would be helpful here.
5. You need to acknowledge the diversity of what could be meant by depression and continue to clarify what you mean by depression when you introduce a study. For instance, are we talking about an MDD diagnosis (or a PDD, or a past MDE) or depression symptoms on a continuum that may or may not reach clinical cutoffs (e.g. CESD).
6. Line 367: 71 months is more than 5 years so you should justify why it was included there are also there are other studies that include older children (such as in line 320).
7. Line 376: It doesn’t seem that the literature is mixed it seems that it is leaning towards there is a not a significant mediation by maternal depression.
8. Line 381: I don’t understand what the first part of the sentence is saying, what is the finding? I think there may be some words missing. Please clarify.
9. Give the acculturation findings their own cohesive paragraph instead of sprinkling them throughout multiple sections to improve clarity.

Discussion

1. Line 430: “contextual stressors in conjunction with depression heightened associations between maternal depression and child maladjustment” is not supported in what you found, you found 2 of these studies were not significant and one was but only when combined with stress and explaining the lack of findings would be helpful.
2. I would tie in the immigrant paradox literature (e.g. Garcia Coll’s work) again to interpret your finding that things get worse with greater acculturation (Lines 485-487). There is a lot of literature on this.
3. Interpret why depression alone did not predict child outcomes as strongly as depression and stress together. For instance this could go after line 468.

Minor points

1. Imprecise language should be specified for instance:
   a. Line 53: should be prevalence rates of major depressive disorder (or whatever you define in your construct) for clarity.
   b. Line 124 these findings are specific to first compared to second generation immigrants not “intergenerational” broadly.
   c. Line 129 should be largest ethnic minority (it isn’t sexual or gender minorities or necessarily racial minority)
