# Supplementary material for: Maternal depression in Latinas and child socioemotional development: A systematic review
Source: PLoS One. 2020 Mar 12;15(3):e0230256. doi: 10.1371/journal.pone.0230256 (PMC7067456; doi:10.1371/journal.pone.0230256)
Supplement: Attachment — Submitted filename: Response_to_reviewers.docx [file pone.0230256.s005.docx]

January 9, 2020

James Swain, M.D., Ph.D., F.R.C.P.C

Academic Editor, PLOS ONE

Dear Dr. Swain,

Please consider the attached manuscript – “Maternal depression in Latinas and child socioemotional development: A systematic review” – for publication in PLOS ONE. This is a revised version of manuscript # **PONE-D-19-24059**. We would like to thank the reviewers for their thoughtful and well-appreciated comments. Below we present the reviewers’ comments and how we addressed them – the line numbers used in our comments are based on the manuscript version with track changes.

| **Reviewer 1 Comments** | |
| --- | --- |
| Given that socioemotional and cognitive outcomes reflect a very wide range of constructs, were there any inclusion criteria for the reliability of measures that were used to study these constructs in young children? | Thank you for this comment. The measures used in the population based studies were all previously validated and counted towards the quality assessment of the study for this systematic review. |
| Was there a requirement for how depression was evaluated and diagnosed in mothers who participated in studies included in this review? | We added this to our Methods, lines 252-257:  Maternal depression could encompass depressive symptoms on a continuum, elevated scores on self-report measures, or clinical diagnosis of a depressive disorder. We did not exclude for co-morbid symptoms or diagnoses such as anxiety, stress, or another form of psychological distress. Self-reported depression measures (e.g. CES-D) often used in population-based studies may more broadly capture psychological distress and diverse affective symptoms (e.g. anxiety) rather than symptoms specific to a depressive disorder. |
| The quality assessment criteria in Table 1 follow the guidelines from the National Heart, Lung and Blood Institute. Are these criteria commonly used to determine the quality of studies assessing mental health outcomes? | These guidelines are used to assess the quality of observational cohort and cross-sectional studies. They are not specific to a particular health condition.  <https://www.nhlbi.nih.gov/health-topics/study-quality-assessment-tools> |
| Related to question 1 above, it is indicated on page 18 of the paper that most studies measured child’s socioemotional development by mother-rated scales? Is it possible that these ratings were affected by the mother’s depression? | Yes, research has shown that mothers with depression may have more negative perceptions of their children. For this reason, three out of five studies using maternal reported child measures in longitudinal studies adjusted for concurrent depression.  In addition, we list this as a limitation of our systematic review results, lines 878-881:  Behavior ratings may be influenced by maternal cultural expectations [20] and maternal depression. Mothers with depression may be more likely to view their children negatively, resulting in potentially biased ratings. |
| More detail would help to understand the quantitative data analytic strategy. Is it possible to obtain the magnitude of effect sizes for the strength of association among the variables of interest? | Thanks for this comment. We clarified that this study is a systematic review, and not a meta-analysis. We were unable to extract reliable effect sizes due to the high heterogeneity of the studies identified. Thus, we decided to present the statistical results, when appropriate, in Table 2, and summarize them in the text. This way, the data can be used in the future when larger and more homogenous literature is available. |
| **Reviewer 2 Comments** | |
| **General Comments** | |
| Use consistent terms for your constructs (many different terms for the outcome throughout) and define your constructs upfront and all that they encompass for clarity.  You say you are only looking at maternal depression but cite a handful that combine stress and depression or depression and other psychopathology. | We defined socioemotional competencies and clarified adverse socioemotional outcomes in the Introduction starting, line 68:  Socioemotional competencies underlie early childhood mental health and encompass “a child’s developing capacity from birth through five years of age to form close and secure adult and peer relationships; experience, regulate, and express emotions in socially and culturally appropriate ways; and explore the environment and learn—all in the context of family, community, and culture.”  …Our review focuses on adverse early social and emotional outcomes associated with maternal depression and greater risk for psychopathology later in life: 1) temperamental traits (e.g., higher negative emotionality and lower self-regulatory capacity) in infants that increase vulnerability to stressful caregiving environments; 2) infant social withdrawal behavior; 3) insecure mother-child attachment; and 4) internalizing (e.g., fearfulness, anxiety) and externalizing (e.g., aggression) behaviors in toddlers and preschoolers.  These outcomes and their measurements are then presented in the results.  We included our maternal depression criteria to our methods and explained why we included studies with comorbid diagnoses in our review, lines 252-257:  Maternal depression could encompass depressive symptoms on a continuum, elevated scores on self-report measures, or clinical diagnosis of a depressive disorder. We did not exclude for co-morbid diagnoses or symptoms such as anxiety, stress, or another form of psychological distress. Self-reported depression measures (e.g. CES-D) often used in population-based studies may more broadly capture psychological distress and diverse affective symptoms (e.g. anxiety) rather than symptoms specific to a depressive disorder. |
| Mediation and moderation are distinct conceptually and have different implications so the many instances of grouping them together is misleading. Set them up separately and interpret them separately since they tell us different things. | We divided moderating and mediating pathways under economic stressors in the Results section where they were previously mixed, lines 647 – 672. |
| **Introduction:**  **Clearly define the literature and your construct for readers to begin with.** | |
| I would strongly recommend narrowing your focus and in particular getting rid of the one study here and there that looks at a different construct entirely than the others (e.g. attachment and psychophysiology). I would recommend focusing on psychological outcomes (social behavior and externalizing) and closely related constructs (e.g. emotion regulation) since the studies seem to overwhelmingly focus on this, it’s much easier to build a cohesive theory around this, and you can clearly define the literature and your construct for readers to begin with. | Thanks for this suggestion. We deleted three cognitive studies (now presented in the supplemental section) and focused on adverse socioemotional outcomes during infancy and early childhood, including insecure attachment due to its integral importance in early social and emotional development.  We decided to keep studies (n=3) using physiological measures since they were included as variables in studies examining children’s behavioral outcomes (except one where cortisol was used as a proxy for infant self-regulation outcomes).  Results, methods, discussion, figures, and tables reflect this modification. |
| What is meant by economic hardship? You go on to describe community violence, overcrowded housing, poverty as variables in the studies so make it clear these are all proxy for poverty that you will be considering under the umbrella of “economic hardship.” | Thank you. We have clarified our use of the term economic hardship, in which variables are used as a proxy for poverty in studies. These are now listed in the Introduction, (lines 141-159) as well as Fig 1:  Poverty-related stressors, which have also been used as a proxy for poverty, encompass numerous risk factors for children: food insecurity, overcrowded housing, and community violence. |
| Restructuring thoughts: Your paper would benefit from an integrated conceptual model presented early on (a figure would be helpful).  It seems that your full model is that acculturative stress and economic stress (poverty) leads to maternal depression which impacts poor child outcomes through compromised parenting.  …then the evidence of the model in white families, then the evidence in LatinX families and the need for research in LatinX families. | Thanks for this great suggestion. We created Fig 1 to show potential pathways by which maternal depression may affect a young child’s (birth to five years old) socioemotional outcomes in the broader context of sociocultural and economic stressor. Chronic maternal stressors may affect the interaction between maternal depression and maternal sensitivity. Such maternal behaviors, in turn, may lead to poor child outcomes.  The Introduction is divided into sections that explain each component of the model: Socioemotional Outcomes, Maternal Sensitivity, Economic and Sociocultural Stressors – and their relationship to Maternal Depression.  In Latinx families, evidence suggests that both economic and cultural stressors are correlated with maternal depression and that maternal depression is correlated to adverse early childhood outcomes. We currently have no sufficiently researched pathways specific to Latinx families delineating how contextual stressors and maternal mental health may be connected to socioemotional child development. Thus, we developed the figure based on the limited evidence and expected pathways. |
| You should comment on the immigrant paradox literature explicitly in the argument for Latinas specifically section. | Thanks for this suggestion. We defined the paradox in the Introduction under the cultural stressors paragraph and indicated its relevance to our review, lines 216-221:  The Latino paradox, wherein less acculturated Latinos experience better health outcomes than more acculturated Latinos, has been documented for maternal and child health: birth weights and breastfeeding rates. However, investigators have not, to our knowledge, explored the relationship between maternal biopsychosocial functioning (e.g., depression) and early child socioemotional outcomes in the context of the Latino paradox. |
| Line 53: comment on depression rates in Latinx populations specifically | We clarified that this rate is specifically for Latina mothers, line 54 - 55:  Prevalence rates of depression (mild to severe) in Latina mothers range from 12 to 59% in the perinatal period, compared to 10 -15% in the general population. |
| Developmental argument: I would recommend adding to your developmental windows section talking about why you chose 0-5 year olds specifically (e.g. include the age ranges in the studies you are citing). What is the evidence (not just the theory) that this range is integral? For example, you could cite here evidence that intervening on child outcomes for 0-5 year olds with depressed mothers improves child psychological outcomes long term. Also explain why you use 5 as a cutoff and not earlier? | Literature wavers between 0-3 and 0-5 to define emerging socioemotional competencies. We quoted a commonly used definition in the Socioemotional Outcomes section, which used the ages 0-5.  We chose 0-5 because parenting and child interventions in preschool age (3-5) have also been shown to improve child trajectories by middle childhood and adolescence. We used an example of empirical evidence under Maternal Sensitivity, lines 133 -136:  Empirical evidence suggests that integrated interventions during the first five years of life to reduce maternal depression and enhance parenting skills may ultimately improve children’s socioemotional developmental trajectories, particularly for the most vulnerable mother-child dyads. |
| Make the argument or mothers vs. fathers. | We included a few sentences on why we focused on mothers rather than fathers, lines 80-82:  We focus on maternal rather than paternal depression due to the high prevalence of maternal depression during sensitive developmental periods and fetal exposure to prenatal depression during pregnancy. |
| Don’t review literature on adolescent outcomes (line 112) if your focus is 0-5 years of age. | Thanks for catching this. We deleted adolescent outcomes. |
| Biology is not “permanent” as you say in line 70. | We reworded this sentence, lines 119-126:  The social and cultural caregiving environment continues to shape a young child’s socioemotional developmental trajectory and, thereby, promotes future resiliency or exacerbates risk for mental health disorders over the life course. |
| **Methods** | |
| Line 157: did you exclude for psychological co-morbid diagnoses (e.g. anxiety etc)? | We did not exclude for comorbid diagnoses. We added this explanation to our methods, lines 253-257:  We did not exclude for co-morbid symptoms or diagnoses such as anxiety, stress, or another form of psychological distress. Self-report depression measures (e.g. CES-D) often used in population-based studies may more broadly capture psychological distress and diverse affective symptoms (e.g. anxiety) rather than symptoms specific to a depressive disorder |
| Line 233: brief description of the three-bag and two bag paradigms would be helpful here. | We wrote a brief description, lines 389-391:  Measures included The Three Bag Task or Two Bag Task, in which the dyad is asked to play with three toys during a 15-minute video-recorded session; trained coders later assess parent behavior. |
| **Results** | |
| You need to acknowledge the diversity of what could be meant by depression and continue to clarify what you mean by depression when you introduce a study. For instance, are we talking about an MDD diagnosis (or a PDD, or a past MDE) or depression symptoms on a continuum that may or may not reach clinical cutoffs (e.g. CESD). | We included what could be meant by depression in our methods, lines 252-253:  Maternal depression could encompass depressive symptoms on a continuum, elevated scores on self-report measures, or clinical diagnosis of a depressive disorder.  Throughout the results, we introduced each study with the specific maternal depression measure, scale of measurement (continuous scores, binary cut-off, clinical diagnosis), and specific child outcome measure. |
| Line 367: 71 months is more than 5 years so you should justify why it was included there are also there are other studies that include older children (such as in line 320). | We kept this study because the majority of children were under five, with a mean of 42 months.  Exposure to maternal depression was before 5 years old (1 and 3.5 years old), so this study fully satisfied the inclusion criteria. The longitudinal nature made this study even stronger. |
| Line 376: It doesn’t seem that the literature is mixed it seems that it is leaning towards there is a not a significant mediation by maternal depression. | Yes, after we deleted academic skills, the FSM studies showed no mediation by maternal depression. We revised, line 641:  …the FSM studies provided no support for the mediating role of maternal depressive symptoms between poverty-related stressors and child outcomes. |
| Line 381: I don’t understand what the first part of the sentence is saying, what is the finding? I think there may be some words missing. Please clarify. | We deleted this part since it is confusing and not necessary. |
| Give the acculturation findings their own cohesive paragraph instead of sprinkling them throughout multiple sections to improve clarity. | The end of our results section has its own acculturation section, beginning on line 673. We included acculturation in our Maternal Sensitivity section because it was imperative to understanding the significant differences in maternal sensitivity between more acculturated and less acculturated mothers, starting on line 557. |
| **Discussion** | |
| Line 430: “contextual stressors in conjunction with depression heightened associations between maternal depression and child maladjustment” is not supported in what you found, you found 2 of these studies were not significant and one was but only when combined with stress and explaining the lack of findings would be helpful.  Interpret why depression alone did not predict child outcomes as strongly as depression and stress together. For instance, this could go after line 468. | We changed the wording, line 733-734:  Maternal stress in conjunction with depression heightened associations between maternal depression and child maladjustment.  We interpreted the lack of findings for the FSM studies and revised the text to address why depression alone did not predict child outcomes as strongly as depression and stress together, starting on line 779:  Perhaps the cumulative effects of chronic stressors are less measurable until later in childhood. The preschool years, however, were the least likely to yield significant associations between maternal depression and child development in our studies unless maternal depression was combined with a measure of stress. Elevated scores on both maternal depression and stress measures may reflect more chronic depressive symptoms, a pattern of depression associated with greater risk for child maladjustment in our longitudinal studies. Moreover, some empirical evidence suggests that maternal stress (e.g., parenting stress, limited social support, marital conflict) may mediate the relationship between maternal depression and behavior problems in toddlers and preschoolers. In immigrant Latino families, economic stressors combined with parental immigration-related stress have predicted increased behavioral difficulties in preschoolers. |
| I would tie in the immigrant paradox literature (e.g. Garcia Coll’s work) again to interpret your finding that things get worse with greater acculturation (Lines 485-487). There is a lot of literature on this. | We cited a book on the immigrant paradox in children. As far as paradox literature, we haven’t found much literature in relation to mental health and young children under five (but added what we found), lines 814-824:  …a study examining the relationship between cultural adaptation in parents and behavioral functioning in preschoolers found that high US identity in Latino parents was associated with more externalizing behaviors, whereas higher ethnic parental identity predicted lower levels of child externalizing and internalizing behaviors. Increased parental acculturation, as measured by language usage, has also been linked to lower (i.e., less adaptive) cortisol responses in preschoolers living in poverty. Although cross-generational research supports an immigrant paradox in behavioral outcomes during adolescence (e.g., substance abuse, teen pregnancy, delinquency), research examining socioemotional outcomes in children under five years of age has been scarce. This is the first review, to our knowledge, to have found support for the Latino paradox in early childhood mental health.  We also included the importance of combining models of both risk and resilience to understand the paradox, line 832:  Maternal biopsychosocial stress across generations stemming from the chronic stressors inherent in unequitable societal structures may be a salient contributing factor; protective cultural factors such as collectivism that confer psychological resilience may also be lost in subsequent generations. Combining models based on risk (e.g., developmental-risk framework) and resilience (e.g., ecocultural theory) may help elucidate underlying mechanisms for the Latino Paradox. |
| **Minor Points:**  Imprecise language should be specified | |
| Line 53: should be prevalence rates of major depressive disorder (or whatever you define in your construct) for clarity.  Line 124 these findings are specific to first compared to second generation immigrants not “intergenerational” broadly.  Line 129 should be largest ethnic minority (it isn’t sexual or gender minorities or necessarily racial minority) | We defined depression to include mild to major depression, line 54-55.  Yes, thank you for catching this. We changed the wording.  Yes, thank you again. We added “ethnic.” |

We are grateful for the opportunity to resubmit this revised manuscript and hope our revisions will be judged satisfactory.

Sincerely,

Hudson Santos, PhD, RN

Assistant Professor

University of North Carolina at Chapel Hill
